# Ginseng Berry Extract Rich in Phenolic Compounds Attenuates Oxidative Stress but not Cardiac Remodeling post Myocardial Infarction

**DOI:** 10.3390/ijms20040983

**Published:** 2019-02-24

**Authors:** Mihir Parikh, Pema Raj, Liping Yu, Jo-Ann Stebbing, Suvira Prashar, Jay C. Petkau, Paramjit S. Tappia, Grant N. Pierce, Yaw L. Siow, Dan Brown, Heather Blewett, Thomas Netticadan

**Affiliations:** 1 Centre for Agri-Food Research in Health and Medicine, St. Boniface Hospital Albrechtsen Research Centre, Winnipeg, MB R2H2A6, Canada; mparikh@sbrc.ca (M.P.); praj@sbrc.ca (P.R.); lyu@sbrc.ca (L.Y.); jstebbing@sbrc.ca (J.-A.S.); sprashar@sbrc.ca (S.P.); jpetkau@sbrc.ca (J.C.P.); gpierce@sbrc.ca (G.N.P.); csiow@sbrc.ca (Y.L.S.); danielbrown500@gmail.com (D.B.); hblewett@sbrc.ca (H.B.); 2 Agriculture and Agri-Food Canada, Winnipeg, MB R2H2A6, Canada; 3 Institute of Cardiovascular Sciences, St. Boniface Hospital Albrechtsen Research Centre, Max Rady College of Medicine, Rady Faculty of Health Sciences, University of Manitoba, Winnipeg, MB R2H2A6, Canada; 4 Department of Food and Human Nutritional Sciences, University of Manitoba, Winnipeg, MB R3T2N2, Canada; 5 Asper Clinical Research Institute & Office of Clinical Research, St. Boniface Hospital, Winnipeg, MB R2H2A6, Canada; ptappia@sbrc.ca; 6 Department of Physiology and Pathophysiology, University of Manitoba, Winnipeg, MB R3E0J9, Canada

**Keywords:** *Panax quinquefolius*, ginseng berry, myocardial infarction, phenolic compounds

## Abstract

The cardioprotective effects of ginseng root extracts have been reported. However, nothing is known about the myocardial actions of the phenolic compounds enriched in ginseng berry. Therefore, this study was undertaken to investigate the effects of American ginseng berry extract (GBE) in an experimental model of myocardial infarction (MI). Coronary artery ligation was performed on Sprague–Dawley male rats to induce MI after which animals were randomized into groups receiving either distilled water or GBE intragastrically for 8 weeks. Echocardiography and assays for malondialdehyde (MDA) and TNF-α were conducted. Flow cytometry was used to test the effects of GBE on T cell phenotypes and cytokine production. Although GBE did not improve the cardiac functional parameters, it significantly attenuated oxidative stress in post-MI rat hearts. GBE treatment also resulted in lower than control levels of TNF-α in post-MI rat hearts indicating a strong neutralizing effect of GBE on this cytokine. However, there was no effect of GBE on the proportion of different T cell subsets or ex-vivo cytokine production. Taken together, the present study demonstrates GBE reduces oxidative stress, however no effect on cardiac structure and function in post-MI rats. Moreover, reduction of TNF-α levels below baseline raises concern regarding its use as prophylactic or preventive adjunct therapy in cardiovascular disease.

## 1. Introduction

Ginseng has been used for centuries in the traditional medicines in Asia. It is a herb derived from genus *Panax*, of family *Araliaceae* and has thirteen different species which are indigenous to Asia and North America [1]. Active components of ginseng include ginsenosides, saponins, polysaccharides, alkaloids, peptides, polyacetylenes, phenolics, and fatty acids [2,3].

Out of all the bioactive compounds of ginseng, ginsenosides have been more extensively studied compared to the phenolic compounds. However, phenolic compounds are now being investigated in several studies for their diverse biological actions [3]. Salicylic acid, p-coumaric acid, ferulic acid, cinnamic acid, and quercetin are some of the phenolic compounds identified in ginseng [3]. Unlike ginseng roots, the chemical composition of ginseng berry is less known. A comprehensive profile of the phenolic compounds found in Korean ginseng berry, root, and leaf has been reported [3]. In the Korean ginseng berry, chlorogenic acid was reported to be the predominant compound present, followed by gentisic acid and rutin [3]. In the North American ginseng berry, caffeic acid and chlorogenic acid were reported to be the active polyphenolic constituents in a study of the protective effect of ginseng berry extract against oxidant injury in cardiomyocytes [4]. In spite of reports of the presence of bioactives in ginseng berry, it is not commercially used and is often discarded as a ‘useless by-products’ [5,6].

A recent study showed that ginseng berry has higher total phenol content (including quercetin, rutin, and resveratrol) than the root [5]. Although both ginseng root and berry have pharmacological actions, in some instances berry has been found to be more effective. Ginseng berry has been reported to have a more potent antihyperglycemic action than the root at the same dose [7]. Ginseng berry has been shown to reduce coagulation of blood [8], improve insulin sensitivity [9], and regulate glucose metabolism [10]. Ginseng berry extract (GBE) was found to protect cardiomyocyte against oxidative stress by activating the antioxidant Nrf2 pathway [11]. An echocardiography study using ginseng root extract demonstrated a significant improvement in left ventricular function [12]. However, ginseng berry with its high phenolic content has not been evaluated for its effect on cardiac structure and function. Accordingly, the present study investigated the effect of a phenolic rich GBE on cardiac structure and function. Furthermore, the damage to the heart muscle resultant from a myocardial infarction (MI) triggers an immune response [13]. When this immune response is uncontrolled it can cause more damage to the heart. Phenolic compounds have been shown to modulate immune responses [14]; but there is a paucity of information on immunomodulatory effects of phenolic extracts from ginseng berry. Thus, our study also assessed immunomodulatory activity of GBE in the myocardial infarction (MI) model induced in rats by coronary artery ligation.

## 2. Results

### 2.1. Phenolic Content and Antioxidant Capacity of GBE

The total phenolic content of the GBE was 3586 ± 04 mg gallic acid equivalents/100 g dry weight using the Folin–Ciocalteu assay. Oxygen radical absorbance capacity (ORAC) assay was performed to assess the oxygen radical scavenging activity of the extract. As expected, GBE exhibited a strong antioxidant capacity with a value of 151,864 ± 883 µmol Trolox equivalents/100 g dry weight. The proximate analysis is presented in Table 1.

### 2.2. Body Weight and Heart Weight Characteristics after MI

Biometrical characteristics were assessed to check whether the coronary artery ligation or treatment with GBE was able to produce any alterations. No significant changes in body weight and heart weight were observed between groups 8 weeks post-surgery. The percentage of scar weight was 21.31% less in GBE-treated MI animals, but not statistically significant when compared to the water-treated MI animals (Table 2). No differences were observed in the heart weight to tibia length ratio values between groups. Similarly, liver and lung weights also did not show significant differences between groups (values not shown).

### 2.3. Lack of Improvement in Cardiac Structure and Function with GBE Treatment

**Figure 1:**
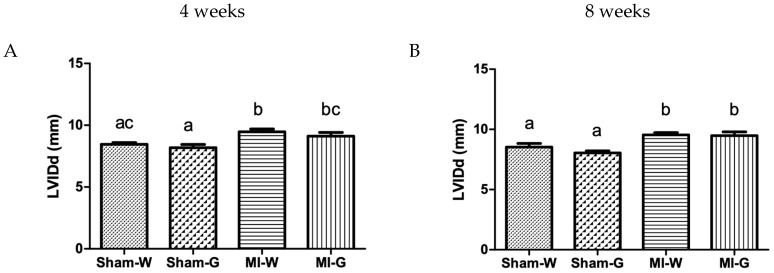

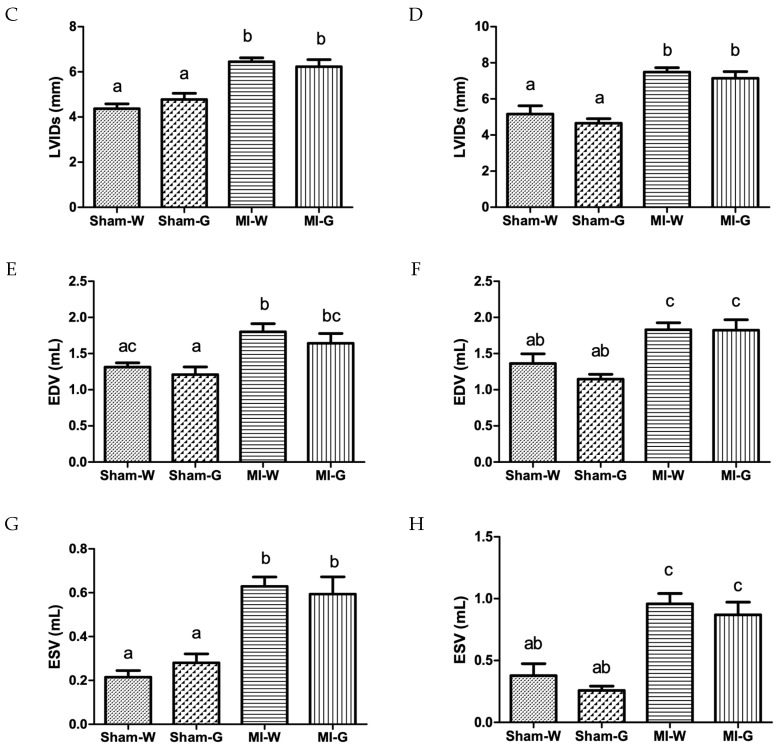
Effect of ginseng berry extract on ventricular chamber dilatation and remodeling. (**A**) LV internal diameter at diastole (LVIDd) at 4 weeks; (**B**) LVIDd at 8 weeks; (**C**) LV internal diameter at systole (LVIDs) at 4 weeks; (**D**) LVIDs at 8 weeks; (**E**) end diastolic volume (EDV) at 4 weeks; (**F**) EDV at 8 weeks; (**G**) end systolic volume (ESV) at 4 weeks; (**H**) ESV at 8 weeks. Values presented are mean ± SEM. Bars with differing letter are significantly different, *p* < 0.05. Sham-W: Sham MI treated with distilled water (*n* = 8); Sham-G: Sham MI treated with GBE 150 mg/kg/body weight/day (*n* = 8); MI-W: MI treated with distilled water (*n* = 12–14); MI-G: MI treated with GBE 150 mg/kg/body weight/day (*n* = 12–14).

M-mode echocardiography was carried out to assess the effect of GBE on the left ventricular remodeling at 4 and 8 weeks post-MI. At 4 weeks, left ventricle (LV) internal diameter (LVID) values at systole and diastole were comparable between water- and GBE-treated sham animals (Figure 1A,C). In contrast, the water-treated and GBE-treated MI groups had significantly higher LVID values when compared to the water-treated sham group indicating left ventricular dilatation (Figure 1A,C). A similar trend was observed at 8 weeks with significantly high LVID values at systole and diastole in the water-treated and GBE-treated MI groups in comparison to water-treated sham group (Figure 1B,D). However, no changes were observed in LVID after GBE treatment in MI group (vs. water treated MI group) at 4 and 8 weeks (Figure 1A–D). LV volumes were also observed to be altered. MI animals treated with water and GBE showed significantly higher end-diastolic volume (EDV) and end-systolic volume (ESV) when compared to water treated sham animals (Figure 1E–H). There was no difference observed between sham animals treated with water and GBE, for EDV and ESV. GBE treatment of MI animals did not produce significant changes in EDV and ESV when compared to the water-treated MI animals (Figure 1E–H).

**Figure 2:**
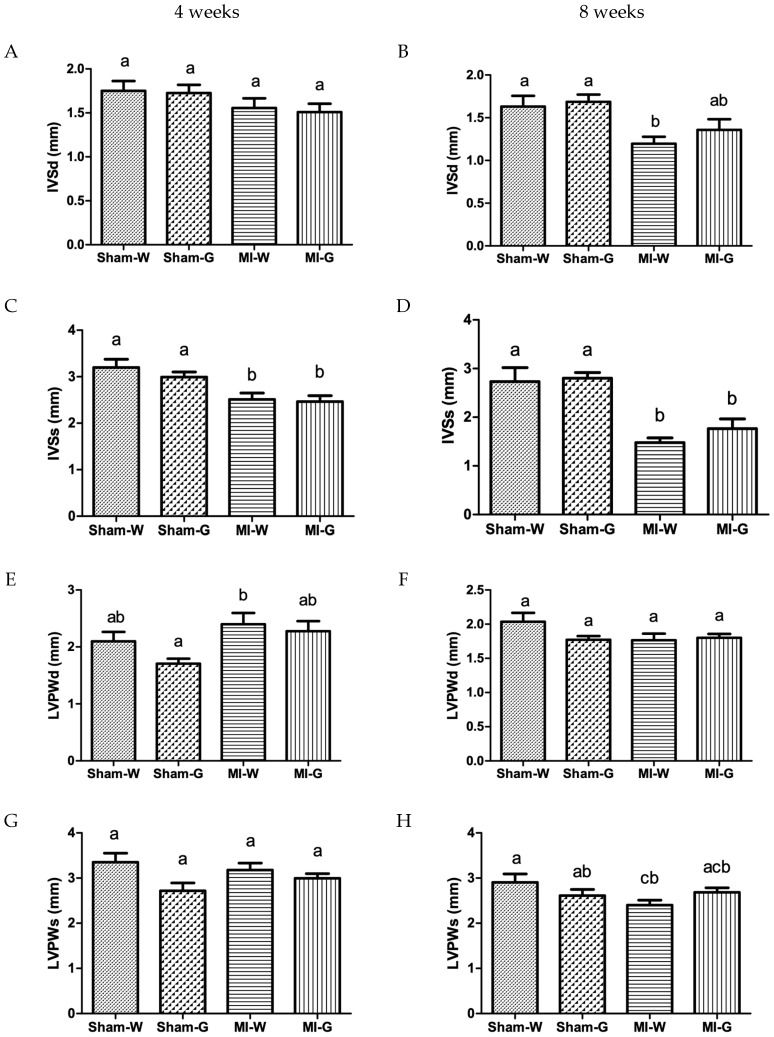
Effect of ginseng berry extract on cardiac hypertrophy. (**A**) Interventricular septal thickness at diastole (IVSd) at 4 weeks; (**B**) IVSd at 8 weeks; (**C**) IVS at systole (IVSs) at 4 weeks; (**D**) IVSs at 8 weeks; (**E**) LV posterior wall thickness at diastole (LVPWd) at 4 weeks; (**F**) LVPWd at 8 weeks; (**G**) LV posterior wall thickness at systole (LVPWs) at 4 weeks; (**H**) LVPWs at 8 weeks. Values presented are mean ± SEM. Bars with differing letter are significantly different, *p* < 0.05. Sham-W: Sham MI treated with distilled water (*n* = 8); Sham-G: Sham MI treated with GBE 150 mg/kg/body weight/day (*n* = 8); MI-W: MI treated with distilled water (*n* = 12–14); MI-G: MI treated with GBE 150 mg/kg/body weight/day (*n* = 12–14).

To check for left ventricular hypertrophy along with the increased LV dilatation, the thickness of interventricular septal thickness (IVS) and LV posterior wall (LVPW) was measured. IVS thickness at systole was observed to be significantly lower at 4 weeks (Figure 2C), and at both systole and diastole at 8 weeks (Figure 2B,D), in the water- and GBE-treated MI animals when compared to water treated sham animals. LVPW thickness values were also found to be significantly low at systole in the water-treated MI group when compared to water treated sham group (Figure 2H). At both 4 and 8 weeks, values for IVS and LVPW thickness were found to be comparable between water and GBE treated MI groups (Figure 2A–H).

**Figure 3:**
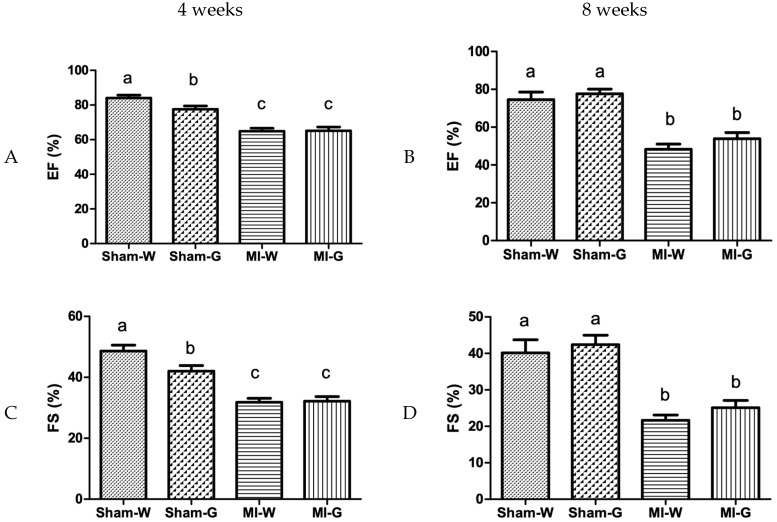
Effect of ginseng berry extract on systolic heart function. (**A**) Ejection fraction (EF) at 4 weeks; (**B**) EF at 8 weeks; (**C**) fractional shortening (FS) at 4 weeks; (**D**) FS at 8 weeks. Values presented are mean ± SEM. Bars with differing letter are significantly different, *p* < 0.05. Sham-W: Sham MI treated with distilled water (*n* = 8); Sham-G: Sham MI treated with GBE 150 mg/kg/body weight/day (*n* = 8); MI-W: MI treated with distilled water (*n* = 12–14); MI-G: MI treated with GBE 150 mg/kg/body weight/day (*n* = 12–14).

Cardiac function was assessed by using M-mode echocardiography. Ejection fraction (EF), a measure of cardiac contraction (systolic heart function), was identical between water- and GBE-treated sham animals. The water-treated and GBE-treated MI groups showed significantly lower EF at 4 and 8 weeks when compared to water treated sham group (Figure 3A,B). EF values were not significantly different between the water- and GBE-treated MI groups (Figure 3A,B). Another parameter of cardiac contraction, fractional shortening (FS), was also significantly reduced in the water- and GBE-treated MI groups when compared to water treated Sham controls (Figure 3C,D). No significant differences were noted for FS values between water and GBE treated MI groups (Figure 3C,D).

**Figure 4:**
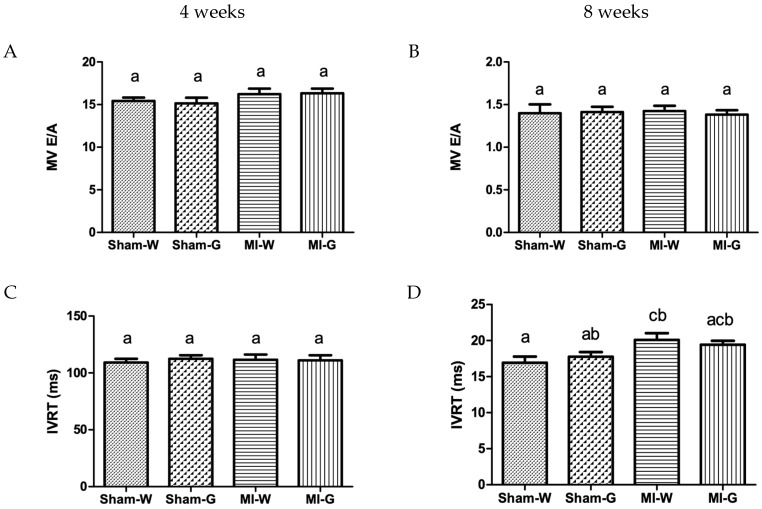
Effect of ginseng berry extract on diastolic heart function. (**A**) Mitral valve E- to A- wave ratio (MV E/A) at 4 weeks; (**B**) MV E/A at 8 weeks; (**C**) Isovolumic relaxation time (IVRT) at 4 weeks; (**D**) IVRT at 8 weeks. Values presented are mean ± SEM. Bars with differing letter are significantly different, *p* < 0.05. Sham-W: Sham MI treated with distilled water (*n* = 8); Sham-G: Sham MI treated with GBE 150 mg/kg/body weight/day (*n* = 8); MI-W: MI treated with distilled water (*n* = 12–14); MI-G: MI treated with GBE 150 mg/kg/body weight/day (*n* = 12–14).

Doppler echocardiography was used to examine the changes in cardiac relaxation (diastolic heart function), post-MI. At 4 and 8 weeks, no changes were observed in the parameters assessing diastolic heart function, mitral valve (MV) E and A wave velocity in all groups (Figure 4A,B). At 4 weeks, the values of another diastolic heart function parameter, Isovolumic relaxation time (IVRT) was also found to be similar in all the groups (Figure 4C). However, IVRT was observed to be significantly increased in the water- and GBE-treated MI groups when compared to water treated sham controls at 8 weeks (Figure 4D). GBE-treated MI animals had values comparable to water-treated MI animals (Figure 4D).

### 2.4. Reduction in Oxidative Stress and Inflammation with Ginseng Berry Extract Treatment

**Figure 5:**
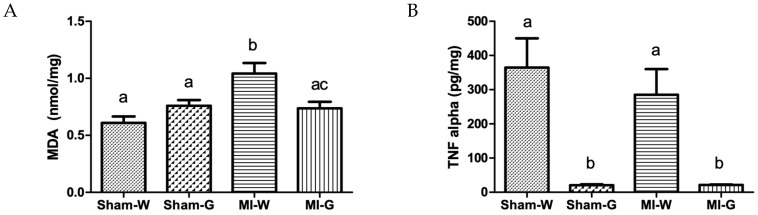
Effect of ginseng berry extract on oxidative stress and inflammation. (**A**) Malondialdehyde (MDA); (**B**) tumor necrosis factor-α (TNF-α). Values presented are mean ± SEM. Bars with differing letter are significantly different, *p* < 0.05. Sham-W: Sham MI treated with distilled water (*n* = 8); Sham-G: Sham MI treated with GBE 150 mg/kg/body weight/day (*n* = 8); MI-W: MI treated with distilled water (*n* = 12–14); MI-G: MI treated with GBE 150 mg/kg/body weight/day (*n* = 12–14).

The 8-week water treated MI animals demonstrated significantly increased MDA levels in the heart tissue when compared to water-treated sham animals (Figure 5A). The GBE treated MI group showed a significant decrease (29%) in MDA levels when compared to the water-treated MI group (Figure 5A). Although, values for TNF-α were comparable between 8-week water treated sham and MI groups, a significant reduction of ~94% was observed in 8-week GBE-treated MI and sham animals when compared to water-treated sham and MI animals, respectively (Figure 5B).

### 2.5. Immune Cell Phenotypes

Using forward vs. side scatter we can identify viable lymphoid cells. Cell shrinkage, one of the first indicators of apoptosis, can be identified by a decrease in forward light scatter. There was no difference among groups in the proportion of cells that appeared in the non-viable lymphoid region.

**Figure 6:**
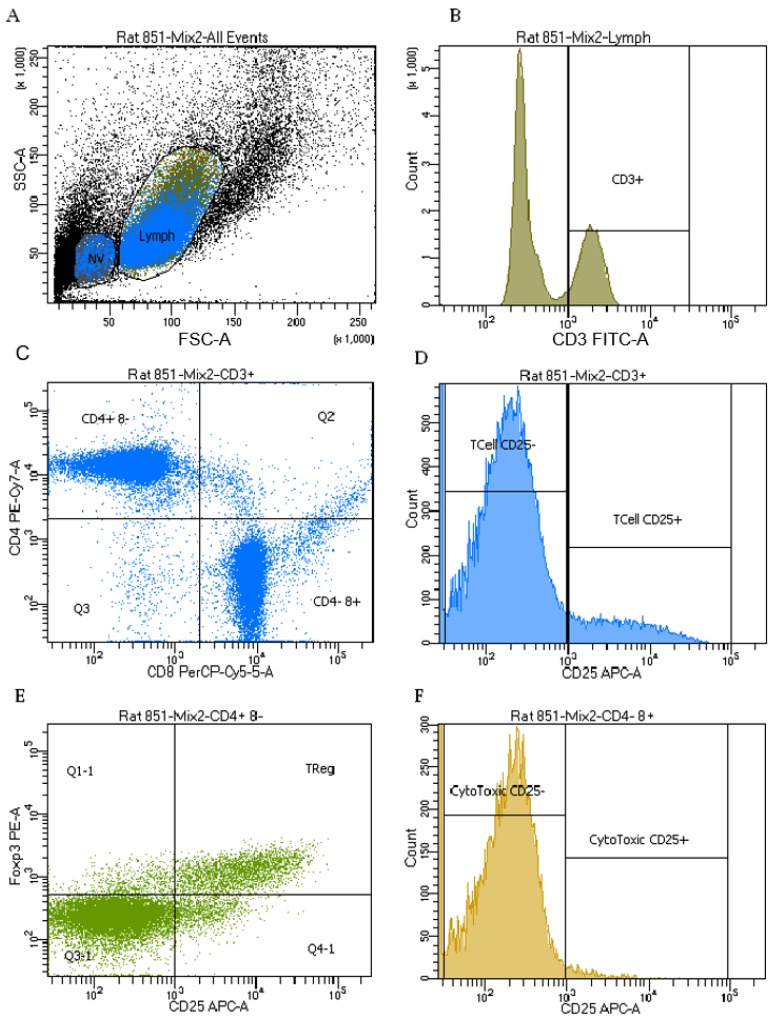
Immune cell phenotyping. (**A**) Definition of viable lymphocytes (Lymph) and non-viable lymphocytes (NV); (**B**) Definition of CD3 binding after gating on Lymph; (**C**) Definition of CD4 and CD8 after gating on CD3^+^ Lymph; (**D**) Definition of CD25 binding after gating on CD3^+^ Lymph; (**E**) Definition of Foxp3 and CD25 after gating on CD3^+^CD4^+^CD8^−^ Lymph; (**F**) Definition of CD25 binding after gating on CD3^+^CD4^−^CD8^+^ Lymph.

In the viable lymphoid region (Figure 6), MI had no effect on the proportion of total T-cells (identified as CD3^+^), helper T-cells (identified as CD3^+^CD4^+^CD8^−^), cytotoxic T-cells (identified as CD3^+^CD4^−^CD8^+^) or activated T-cells (identified as CD3^+^CD25^+^) and activated cytotoxic T-cells (identified as CD3^+^CD4^−^CD8^+^CD25^+^). MI did result in an 8.0% lower proportion of activated helper T-cells (identified as CD3^+^CD4^+^CD8^−^CD25^+^) and 9.8% lower proportion of T-regulatory cells (identified as CD3^+^CD4^+^CD8^−^CD25^+^foxp3^+^) compared to sham-operated rats. GBE treated rats had a 9.4% higher proportion of T-cells and 13.2% higher proportion of cytotoxic T-cells, but a 7.4% lower proportion of helper T-cells compared to water treated rats. There was no effect of GBE on the proportion of activated T-cells, activated helper T-cells, activated cytotoxic T-cells and T-regulatory cells (Table 3).

**Figure 7:**
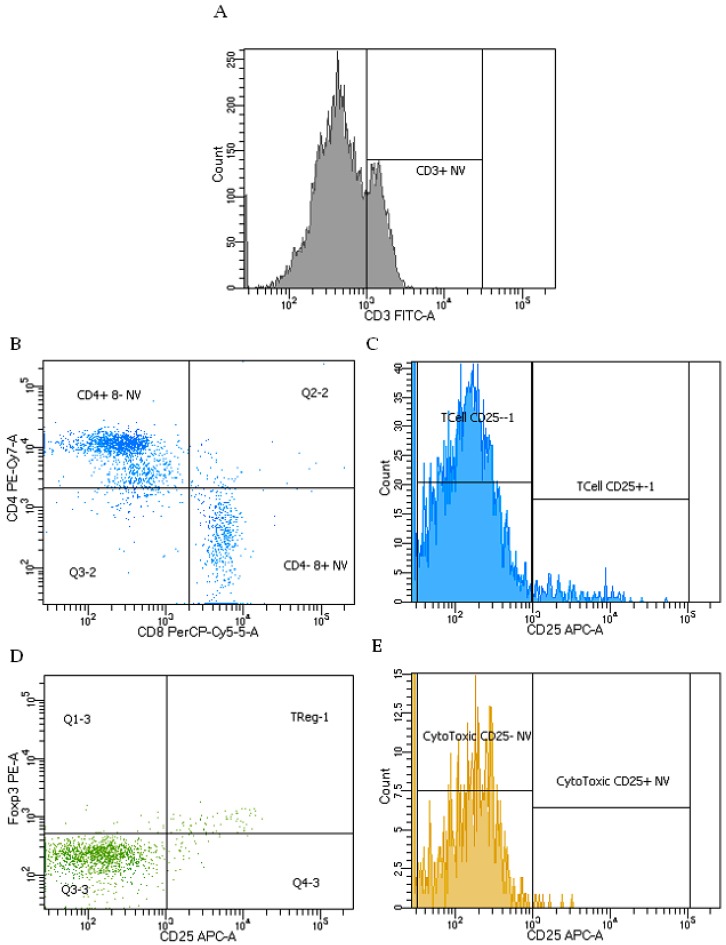
Representative flow cytometry plot for non-viable lymphocytes. (**A**) Definition of CD3 binding after gating on non-viable lymphocytes (NV); (**B**) Definition of CD4 and CD8 after gating on CD3^+^ NV; (**C**) Definition of CD25 binding after gating on CD3^+^ NV; (**D**) Definition of Foxp3 and CD25 after gating on CD3^+^CD4^+^CD8^−^ NV; (**E**) Definition of CD25 binding after gating on CD3^+^CD4^−^CD8^+^ NV.

In the non-viable lymphoid region compared to the viable lymphoid region, there were lower proportions of all T-cell phenotypes, except for helper T-cells (Figure 7). Rats that had an MI had a 30% lower proportion of activated cytotoxic T-cells compared to sham-operated rats in the non-viable region. There was a trend towards a lower proportion of T-regulatory cells after MI compared to sham (*p* = 0.06), but no significant difference in the proportion of total T-cells, activated T-cells, helper T-cells, activated helper T-cells, or cytotoxic T-cells in the non-viable region. Compared to water-treated controls, rats treated with GBE had a 29.4% higher proportion of T-cells and a 10% lower proportion of activated Helper T-cells in this region. There was no difference in the proportion of activated T-cells, helper T-cells, T-regulatory cells, cytotoxic T-cells and activated cytotoxic T-cells (Table 3).

### 2.6. Cytokines Production

ConA stimulated splenocytes from rats that had an MI when compared to controls, did not show a significant difference in IL-2, IFNγ, IL-10 or TNFα concentrations produced or between GBE treatment and control (Table 4).

Unstimulated samples were below the lower limit of detection for IFNγ (6.8 pg/mL) and TNFα (27.7 pg/mL). Unstimulated concentrations of IL-2 and IL-10 were not affected by MI or GBE treatment (Table 4).

## 3. Discussion

The present study is the first to report that ginseng berry phenolic extract has a strong antioxidant effect despite no effect on cardiac structure and function in the condition of a MI.

Although there is no information in the literature on the effects of ginseng berry on heart structure and function in any settings of cardiovascular disease, some studies conducted with Korean ginseng (*Panax ginseng*) have shown that GBE exerts anti-atherosclerotic [15], anti-diabetic, and anti-obesity effects [16]. Unlike ginseng root extract, GBE has been found to inhibit the mRNA expression of interleukins 1β and 6 inflammatory markers and it is likely through this mechanism that it exerts its antihyperglycemic and anti-obesity effects [17]. GBE also increases prothrombin time, indicating potential atherothrombotic effects [8].

Ginseng consists of phenolic compounds which include phenolic acids and flavonoids with strong antioxidant activity, but these are less characterized as compared to the ginsenosides. Chung et al. reported 4–9-fold higher phenolic compounds in the 3–6 year-old Korean ginseng berry (*Panax ginseng*) compared to the same age roots [3]. A higher phenolic content has been observed in the older Korean ginseng berries when compared to younger ones; the total amount increasing by 20–48% with an increase in the cultivation year. DPPH free-radical-scavenging activity (DPPH activity) of the 3–6 years old Korean ginseng berry is 3–5 fold higher than the root, suggesting the strong antioxidant activity of the berry [3]. In this context, an extract derived from American ginseng berries protected cardiomyocytes from oxidative stress induced by H_2_O_2_ and antimycin A [8]. An approximately 60% reduction in cardiomyocyte death was observed with the American GBE, clearly demonstrating that it can salvage cardiomyocytes from the oxidative injury [18]. The proposed mechanisms for the American GBE antioxidant actions included direct free radical scavenging activity and stimulation of NO synthesis [18]. This study, therefore, examined the antioxidant potential of North American GBE (*Panax quinquefolius*) in addition to examining its cardioprotective potential in an experimental model of MI.

The present study demonstrated a significant reduction in ventricular function after a MI. The reasons behind this adverse outcome include an impaired contractile function as well as infarct expansion leading to ventricular dilatation. Two-dimensional echocardiography performed on patients with an acute transmural MI has demonstrated dilatation of the infarct zone. This infarct expansion phase, which occurs due to the slippage of necrotic myocardial fibers, starts as early as 3 days after infarction [19]. This early regional dilation occurring in the infarcted zone results in an overall left ventricular dilatation. The ventricular dilatation may continue until 30 months after infarction. Unlike the early expansion phase, both infarcted and non-infarcted segments are affected during the chronic phase dilation [20]. This disproportionate cardiac dilatation alters left ventricular topography and is associated with poor prognosis for long-term survival of MI patients [19]. Left ventricular dilatation has been associated with the progression of cardiac dysfunction as cardiac and stroke indices decrease. Of note, ventricular dilatation results in and is not the result of deteriorating cardiac function [21]. Left ventricular function is usually assessed by ejection fraction and end diastolic and systolic volumes. Ejection fraction has been observed to have a strong effect on mortality. With the increase in left ventricular internal diameter, there are substantial increases in end systolic and diastolic volumes with consequently increased stress on ventricular wall. Earlier studies with gated scintiphotography revealed an increase in left ventricular end-systolic volume and decrease in ejection fraction in MI patients compared to normal subjects [22]. Three months follow-up demonstrated increased values of LV ejection fraction in patients with clinical improvement and decreased values with clinically worsened cases [22]. Moller et al demonstrated that echocardiographically determined ejection fraction was a powerful predictor of mortality during a median follow up of 19 months [23]. A progressive increase was noted in the cardiac mortality in patients with ejection fraction below 40% [24]. Another important determinant of the ventricular function post-MI is the end systolic volume (ESV). Consideration of ESV along with EF adds more predictive power for mortality risk stratification of MI patients [25]. Echocardiographic analysis revealed that GBE treatment does not prevent infarct-related impairment in cardiac structure and function.

Reactive oxygen species produced during oxidative stress damage membrane lipids, proteins, DNA thereby causing apoptosis of cardiomyocytes and eventually resulting in cardiac dysfunction [26]. Infiltration of inflammatory cells in the myocardium after an ischemic event initiates an exaggerated inflammatory response, this further accelerates and worsens ventricular remodeling by increasing myocardial injury [27]. Higher levels of the inflammatory marker TNF-α have been associated with ventricular dilation and cardiac fibrosis. Our results demonstrate a significant decrease in the LV levels of TNF-α and MDA due to GBE treatment, indicating that it can reduce the inflammatory and oxidative response in MI. The substantial attenuation of cardiac oxidative stress observed in MI rats could be associated with the robust antioxidant capacity of GBE, as determined by its oxygen radical scavenging activity. Despite exerting strong antioxidant effects, however, GBE was unable to ameliorate cardiac remodeling and rescue cardiac function in MI rats.

The large GBE mediated decline in cardiac inflammatory response observed in the present study is consistent with a previous report which showed that treatment with Korean ginseng berry (*Panax ginseng*) suppressed the expression of TNF-α and thereby reduced atherosclerotic lesions [15]. Chronic use of TNF-α blockers has nevertheless been associated with increased risk of cardiovascular complications. A deficiency in TNF signaling for an extended period of time can cause immune system defects. The cardiac effects of TNF-α are biphasic. While high levels of TNF-α is associated with apoptosis, the basal level of TNF-α is required to maintain cytoprotective Nrf2 signaling [28]. Kurrelmeyer et al reported the importance of TNF signaling in protecting cardiomyocytes against ischemic injury [29]. The protective mechanism against hypoxic damage could be via activation of protein kinase A which stimulates SERCA2a thereby reducing intracellular calcium concentration during calcium overload [30]. Thus, TNF-α signaling helps in pumping calcium ions in the sarcoplasmic reticulum and restoring cytosolic calcium back to baseline levels [30]. It is possible that the reduction of TNF-α levels due to GBE observed in the present study may be as a consequence of: (a) reduced expression of the TNF-α gene and a lessened production of TNF-α protein, or, (b) increased production of soluble TNF-α receptors, which could bind and inactivate TNF-α [31]. However, the results of our examination of T cell phenotypes and function and ex-vivo cytokine production showed no differences in the immune response to GBE.

### Limitations and Future Opportunities

This study has certain limitations that should be taken into account while interpreting the observed findings. Despite the study being sufficiently powered for analysis, the sample size may have been too small as the observed effect size was small. A preliminary pharmacokinetic study would have assisted in appropriate dose selection and treatment regimen. In addition, more than once daily administration might have resulted in a sufficient plasma steady state concentration that could have produced a different outcome. Furthermore, pretreatment with GBE may have resulted in an adequate cardiac tissue distribution of polyphenols necessary for potentiating the endogenous antioxidant system during an ischemic insult. The results of our study open up opportunities for further investigation including a comparative study of a polyphenol rich extract from ginseng berries vs. polyphenol rich extract from roots, and other parts of the plant.

## 4. Materials and Methods

### 4.1. Ginseng Berry Pulp Extract

Ginseng berries from three-year-old *Panax quinquefolius* L. (North American ginseng) were provided by C & R Atkinson Farms Ltd, St. Williams, ON, Canada. The berries were stored at −20 °C until they could be freeze-dried in smaller batches. After freeze-drying berries, the seeds were removed and the pulp was ground to a fine powder. Pulp extracts were prepared in batches at a ratio of 10 g ground pulp per 200 mL of 80% methanol. The slurry was mixed at room temperature using a rotary shaker, at 200 rpm for 1.5 h. The mixture was centrifuged at room temperature for 15 min at 10,000× *g*, saving the pellet for further extraction. The supernatant was collected and stored at −20 °C and the remaining pulp went through two additional rounds of extraction with 80% methanol. All supernatants were pooled together and stored at −20 °C. The solvent was removed by rotary evaporation and the remaining aqueous extract was freeze-dried. Multiple extracts were performed to produce sufficient ginseng berry pulp extract for the study. Dried extracts were further ground to a fine powder and combined to form a homogeneous product for the study. The pH of the dried extract was 4.5 and the final product was stored at −80 °C

### 4.2. Quantification of Phenolic Content and Antioxidant Activity

To ensure complete mixing of dried samples three biological replicates were prepared. A quantity of 0.125 g dried powder of each of these replicates (a, b, and c) was dissolved in 3 mL of autoclaved 18 Ώ Milli-Q water. Tubes were vortexed, sonicated for 10 min and then centrifuged at 10,000× *g*. The supernatant was used for subsequent measurement. The assay was performed in a 96 well plate by combining reagent and sample in sodium carbonate buffer. By using a protocol modified from Ainsworth and Gillespie [32], the total phenolic content was determined by spectrometry using SpectraMax M5 (Molecular Devices, San Jose, CA, USA) microplate reader at 765 nm wavelength. A method modified from Gillespie et al [33] was used to determine the oxygen radical absorbance capacity (ORAC) using 2,2-azobis(2-amidinopropane) dihydrochloride (Wako Chemicals, Richmond, VA, USA) as a peroxyl generator. The decline in fluorescence of fluorescein was measured kinetically for 60 min using a SpectraMax M5 microplate reader and the area under the curve was calculated for each sample.

### 4.3. Proximate Analysis

The core proximate and dietary chemical composition of the sample was determined using standard AOAC methods for ash (AOAC 923.03), fat (AOAC Am5-04), crude fiber (AOAC Ba 6a-05), crude protein modification (AOAC 990.03) and moisture (AOAC 930.15). All tests were conducted by the Central Testing Laboratory (Winnipeg, MB, Canada).

### 4.4. Animal Study

This study protocol was approved by the University of Manitoba Office of Research Ethics and Compliance and Animal Care Committee and was done in accordance with the guidelines by the Canadian Council for Animal Care. Male Sprague Dawley rats (150–175 g; Charles River Laboratories, Quebec, Canada) were housed in a temperature and humidity controlled room with a 12 h light/dark cycle. Rats were anesthetized with 1–5% isoflurane with oxygen at a flow rate of 2 L min^−1^ and kept in a surgical plane of anesthesia with 2% isoflurane during surgery and subjected to permanent ligation of the left anterior descending artery (LAD) to induce MI or to sham surgery. A left thoracotomy was done, and the heart was gently exposed from the pericardial sac through the incision. The left anterior descending coronary artery (LAD) was located and occluded with 6-0 polypropylene silk suture at about 2 mm from aortic root. The suture was tied and the ligation was estimated to be successful when the anterior wall of the left ventricle turned pale. The heart was repositioned, then chest compressed to remove any air from the cavity and the incision was closed using a purse string suture. Sham-operated animals that served as normal control were subjected to similar surgical procedures except that the LAD was not ligated. Buprenorphine 0.05 mg kg^−1^ was administered pre- and post-surgery (2 times a day for 2 days) subcutaneously as an analgesic agent to all rats. All surviving sham and MI rats were assigned to the following 4 treatment groups: (1) Sham MI—distilled water as vehicle (Sham-W); (2) MI—distilled water as vehicle; (MI-W); (3) Sham MI—ginseng berry extract 150 mg/kg/body weight/day (Sham-G); (4) MI—ginseng berry extract 150 mg/kg/body weight/day (MI-G). The 4 groups consisted of sham (*n* = 8) and MI (*n* = 12–14) rats and received the treatments by oral gavage for 8 weeks. The sample size was calculated using G*Power statistical software with the power of study kept at 80%. Animals were regularly monitored for well-being.

#### 4.4.1. Transthoracic Echocardiography

All experimental rats were weighed and anesthetized with 3% isoflurane in a chamber, and then kept under 1.5–2% isoflurane throughout the procedure. An echocardiogram was obtained at 4 and 8 weeks post-surgery by 2D guided M-mode and Doppler modalities with a 13 MHz probe (Vivid E9; GE Medical Systems, Milwaukee, WI, USA) as described by us earlier [34]. 2D M-mode parasternal short-axis view images were obtained to determine systolic functional parameters such as the percentage of left ventricle (LV) ejection fraction (EF), fractional shortening (FS), and end-systolic volume (ESV) and end-diastolic volume (EDV). Doppler measurements included isovolumic relaxation time (IVRT), mitral valve (MV) E wave, A wave, and E wave deceleration time. The cardiac structural parameters such as interventricular septal thickness (IVS), LV posterior wall thickness (LVPW), and LV internal diameter (LVID) at diastole and systole were determined from parasternal short-axis view images. All echocardiographic images were analyzed to calculate the listed parameters using EchoPAC software (GE Medical Systems, Milwaukee, WI, USA). All measurements were performed and averaged from three cardiac cycles to account for interbeat variability.

#### 4.4.2. Biological Sample Collection and Analysis

All animals were anesthetized with ketamine/xylazine (9.0 mg per 100 g and 0.9 mg per 100 g IM). The depth of anesthesia was assessed by pedal withdrawal reflex. The blood sample was collected from the inferior vena cava by opening the thoracic cavity and the heart was immediately excised. The whole heart was rinsed in PBS, LVs, septum, and fibrotic scar tissues were separated, weighed and flash frozen in liquid nitrogen.

Percentage of infarct (scarred/fibrotic) LV tissue was calculated by dividing the weight of scarred LV tissue by whole weight of LV tissue as described previously [35]. Evidence of overt heart failure was assessed by determining the presence of ascites and by calculating the lung and liver wet-to-dry weight ratio in all rats.

To determine MI-associated oxidative stress and inflammation, the levels of the lipid peroxidation product, malondialdehyde (MDA), and tumor necrosis factor-α (TNF α) as a proinflammatory marker were assessed in the heart tissue using commercial kits.

### 4.5. T-cell Phenotypes and Function

To assess the effect of MI and GBE treatment on T-cell phenotypes and activation state and T-cell function, the following experiments were conducted.

#### 4.5.1. Isolation of T-cells

Single cell suspensions were obtained by pressing spleens through a 100µm cell strainer using the barrel of a sterile syringe into a sterile Petri dish containing Hank’s buffered saline supplemented with 10 mM-HEPES, 4% fetal bovine serum, and 1% antibiotic/antimycotic at pH 7.4. Erythrocytes were lysed with ammonium chloride buffer. Cells were subsequently washed and re-suspended in label buffer (PBS containing 23 mM-sodium azide and 2% fetal bovine serum). Cell count and viability was completed using a Nexcelom AutoT4Plus. Cell concentration was adjusted to 1 × 10^7^ cells/mL in label buffer [36].

#### 4.5.2. Cytokine Determination

Splenocytes were resuspended in RPMI-1640 supplemented with 10mM-HEPES, 10 mM-sodium bicarbonate, 1 mM-sodium pyruvate, 2 mM-gluatmine, 0.1 mM-non-essential amino acids, 50 µM-2-mercatpoethanol, 1% antibiotic/antimycotic and 5% fetal bovine serum at pH 7.2 at a concentration of 1 × 10^6^ splenocytes/mL and incubated at 37 °C and 5% CO_2_ with 2.5 µg/mL Concanavalin A (ConA), or unstimulated for 48 hours. After incubation, samples were centrifuged at 400 g at 4 °C for 5 min to pellet cells. Supernatants were stored at −80 °C until analysis. The concentration of IL-2 (lower limit of detection (LLOD) 0.46 pg/mL), IFNγ (LLOD 6.8 pg/mL), IL-10 (LLOD 19.4 pg/mL), and TNFα (LLOD 27.7 pg/mL) were measured in cell culture supernatants using a cytometric bead array on a FACSCanto II flow cytometer. All samples were analyzed in duplicate with CV <10% according to the BD Cytometric Bead Array Mouse/Rat Soluble Protein Master Buffer Kit Instruction Manual [37].

#### 4.5.3. Phenotyping

T-cell phenotypes were determined by flow cytometry using isolated splenocytes. Monoclonal antibodies against rat CD3 (FITC label, clone 1F4, isotype mouse IgM_κ_), CD4 (PE-Cy7 label, clone OX-35, isotype mouse IgG_2a,κ_), CD8 (PerCP label, clone OX-8, isotype mouse IgG_1,κ_), CD25 (APC label, clone OX39, isotype Mouse IgG_1,κ_), foxp3 (PE label, clone FJK-16s, isotype rat IgG_2a,κ_) were obtained from BD BioSciences (Mississauga, ON, Canada). Antibodies were incubated with 1 × 10^6^ cells/mL for 30 min at 4 °C in the dark. Cells were washed, and then incubated in Foxp3 fixation/permeabilization working solution at 4 °C for 18 h. Following incubation cells were washed with the permeabilization buffer and incubated with mouse CD16/CD32 antibody at 4 °C for 5 min to block non-specific binding. Foxp3 was added to cells and incubated at 4 °C in the dark for at least 30 min. Finally, treated cells were washed using the permeabilization buffer and re-suspended in PBS. Data were acquired on a FACSCanto II flow cytometer using the 488 nm and 633 nm lasers. Figure 6 shows representative flow cytometry plots. Forward versus side-scatter plots were used to gate on intact lymphoid cells and non-viable cells. The data were collected in list-mode format with the analyses based on 100,000 cells satisfying the light scatter gate for lymphocytes using Cell Diva software (v8.0.1). Unstained cells were used to assess auto-fluorescence, isotype controls to assess background staining, and single color samples were employed to adjust color compensation [36].

### 4.6. Statistical Analysis

All values are represented as means ± SEM. Two-way analysis of variance (ANOVA) test was used to determine the effect of surgery (factor 1) and treatment (factor 2) and their interaction (SAS, version 9.4, SAS Institute, Cary NC, USA). Unpaired t-test was utilized for comparison between 2 groups Significant differences among means were determined using LSmeans. Differences were considered significant at *p* ≤ 0.05.

## 5. Conclusions

The results of this study suggest that although GBE exerted potent antioxidant activity, it was unable to recover cardiac function in post-MI rats.

## Figures and Tables

**Table 1:** Proximate analysis of ginseng berry extract.

Constituent	Value (%)
Moisture	8.19
Dry matter	91.81
Crude protein	8.61
Crude fibre	1.54
Fat	0.90
Ash	12.50
Non-fibre carbohydrates	68.26
Total digestible nutrients	69.77

**Table 2:** Biometrical characteristics of sham and MI animals with and without ginseng berry extract treatment for 8 weeks after coronary artery ligation.

Parameter	Sham-W	Sham-G	MI-W	MI-G
Body weight (g)	573.1 ± 11	579.9 ± 21	538.4 ± 12	549 ± 16
Heart weight (g)	1.433 ± 0.05	1.355 ± 0.05	1.458 ± 0.03	1.415 ± 0.05
LV weight (g)	0.70 ± 0.060	0.86 ± 0.051	0.73 ± 0.03	0.82 ± 0.04
% infarct	-	-	32.37 ± 2.8	25.47 ± 2.3
Heart weight/Tibia length (g/cm)	0.32 ± 0.009	0.30 ± 0.01	0.33 ± 0.0	0.31 ± 0.0
LV weight/Tibia length (g/cm)	0.15 ± 0.013	0.19 ± 0.011	0.16 ± 0.01	0.18 ± 0.01

Values presented are mean ± SEM. MI: Myocardial infarction; LV: Left Ventricle; Sham-W: Sham MI treated with distilled water (*n* = 8); Sham-G: Sham MI treated with GBE 150 mg/kg/body weight/day (*n* = 8); MI-W: MI treated with distilled water (*n* = 12–14); MI-G: MI treated with GBE 150 mg/kg/body weight/day (*n* = 12–14).

**Table 3:** T-cell phenotypes in viable and non-viable gates.

Lymphocyte Phenotypes	Model			Treatment			Interaction				
	Sham *n* = 16	MI *n* = 23	*p*-Value	Water *n* = 20	GBE *n* = 19	*p*-Value	Sham Water *n* = 8	Sham GBE *n* = 8	MI Water *n* = 12	MI GBE *n* = 11	*p*-Value
**Viable Lymphocytes**											
CD3^+^ % lymphocytes	30.0 ± 0.9	30.0 ± 0.8	0.99	28.7 ± 0.8	31.4 ± 0.9	0.03	29.2 ± 1.3 ^ab^	30.9 ± 1.3 ^ab^	28.2 ± 1.0 ^b^	31.9 ± 1.1 ^a^	0.39
^1^ CD25^+^ % of CD3^+^	11.5 ± 0.3	10.9 ± 0.3	0.14	11.5 ± 0.3	10.9 ± 0.3	0.15	11.7 ± 0.5	11.3 ± 0.4	11.3 ± 0.4	10.4 ± 0.4	0.65
CD4^+^CD8^−^ % of CD3^+^	57.1 ± 1.2	57.5 ± 1.0	0.80	59.5 ± 1.1	55.1 ± 1.1	0.01	58.7 ± 1.7 ^a^	55.5 ± 1.7 ^ab^	60.4 ± 1.4 ^a^	54.6 ± 1.5 ^b^	0.41
^2^ CD25^+^ % of CD3^+^CD4^+^CD8^−^	16.3 ± 0.4	15.0 ± 0.4	0.04	15.9 ± 0.4	15.4 ± 0.4	0.40	16.7 ± 0.6 ^a^	15.9 ± 0.6 ^ab^	15.1 ± 0.6 ^ab^	14.9 ± 0.6 ^b^	0.54
^3^ Foxp3^+^CD25^+^ % of CD3^+^CD4^+^CD8^−^	12.3 ± 0.4	11.1 ± 0.3	0.03	11.9 ± 0.4	11.6 ± 0.4	0.60	12.8 ± 0.5 ^a^	11.8 ± 0.5 ^ab^	10.9 ± 0.5 ^b^	11.4 ± 0.5 ^ab^	0.17
CD4^−^CD8^+^ % of CD3^+^	39.6 ± 1.2	39.2 ± 1.0	0.80	37.0 ± 1.1	41.9 ± 1.1	0.004	37.8 ± 1.7 ^ab^	41.5 ± 1.7 ^a^	36.2 ± 1.4 ^b^	42.3 ± 1.5 ^a^	0.46
^4^ CD25^+^ % of CD3^+^CD4^−^CD8^+^	2.8 ± 0.2	2.7 ± 0.1	0.66	2.7 ± 0.2	2.7 ± 0.1	0.97	2.7 ± 0.2	2.9 ± 0.2	2.8 ± 0.2	2.6 ± 0.2	0.30
**Non-Viable lymphocytes**											
CD3^+^ % non-viable cells	27.2 ± 1.1	25.7 ± 0.9	0.31	23.1 ± 1.0	29.9 ± 1.0	<0.0001	22.9 ± 1.5 ^b^	31.5 ± 1.5 ^a^	23.2 ± 1.3 ^b^	28.2 ± 1.4 ^a^	0.22
^5^ CD25^+^ % of CD3^+^	4.9 ± 0.2	4.3 ± 0.2	0.07	4.9 ± 0.2	4.3 ± 0.2	0.06	4.9 ± 0.3 ^a^	4.8 ± 0.3 ^a^	4.8 ± 0.3 ^a^	3.9 ± 0.3 ^b^	0.17
CD4^+^CD8^−^ % of CD3^+^	75.6 ± 1.3	74.4 ± 1.1	0.49	74.9 ± 1.1	75.1 ± 1.2	0.94	75.7 ± 1.8	75.5 ± 1.8	74.2 ± 1.5	74.7 ± 1.6	0.83
^6^ CD25^+^ % of CD3^+^CD4^+^CD8^−^	4.9 ± 0.2	4.5 ± 0.2	0.13	4.9 ± 0.2	4.4 ± 0.2	0.05	4.9 ± 0.3 ^a^	4.9 ± 0.3 ^a^	5.0 ± 0.2 ^a^	4.0 ± 0.2 ^b^	0.08
^7^ Foxp3^+^CD25^+^ % of CD3^+^CD4^+^CD8^−^	3.1 ± 0.1	2.7 ± 0.1	0.06	3.0 ± 0.1	2.8 ± 0.1	0.18	3.2 ± 0.2 ^a^	3.0 ± 0.2 ^ab^	2.9 ± 0.2 ^ab^	2.6 ± 0.2 ^b^	0.59
CD4^−^CD8^+^ % of CD3+	17.5 ± 0.9	18.3 ± 0.8	0.56	17.3 ± 0.9	18.5 ± 0.9	0.36	16.7 ± 1.3	18.4 ± 1.3	18.0 ± 1.1	18.6 ± 1.2	0.66
^8^ CD25^+^ % of CD3^+^CD4^−^CD8^+^	2.3 ± 0.3	1.6 ± 0.2	0.05	2.2 ± 0.2	1.7 ± 0.2	0.17	2.4 ± 0.4 ^a^	2.2 ± 0.4 ^ab^	1.9 ± 0.3 ^ab^	1.2 ± 0.3 ^b^	0.54

Values are means (pg/mL) ± standard error. Means with different superscript letters are significantly different (*p* < 0.05). GBE: Ginseng berry extract. ^1,4^: Outliers (value >3× standard deviation of mean) were removed: 2 from MI water and 1 from sham water group; ^2,3,7^: Outliers (value >3× standard deviation of mean) were removed: 2 from MI water; ^5,6,8^: Outlier (value >3× standard deviation of mean) were removed: 1 from MI water.

**Table 4:** Supernatant cytokine concentrations from concanvalin A-stimulated and unstimulated splenocytes.

Cytokines	Model			Treatment			Interaction				
	Sham *n* = 16	MI *n* = 23	*p*-Value	Water *n* = 20	GBE *n* = 19	*p*-Value	Sham Water *n* = 8	Sham GBE *n* = 8	MI Water *n* = 12	MI GBE *n* = 11	*p*-Value
IL-2 ConA	2111 ± 181	1890 ± 79	0.24	1910 ± 133	2055 ± 114	0.47	2067 ± 279	2155 ± 248	1805 ± 125	1983 ± 90	0.80
^1^ IL-2 UNS	3.4 ± 0.2	4.0 ± 0.4	0.35	3.9 ± 0.4	3.4 ± 0.3	0.52	3.5 ± 0.6	3.3 ± 0.2	4.2 ± 0.6	3.6 ± 0.5	0.72
^2^ IFNγ ConA	383.0 ± 75	267.0 ± 35	0.13	312.9 ± 59	312.7 ± 47	0.79	427.8 ± 131	343.7 ± 89	245.9 ± 49	290.1 ± 51	0.40
IFNγ UNS	ND	ND	/	ND	ND	/	ND	ND	ND	ND	/
^3^ IL-10 ConA	49.2 ± 3.3	47.3 ± 3.1	0.65	48.4 ± 4.1	47.9 ± 2.3	0.81	51.9 ± 5.4	46.9 ± 4.2	45.9 ± 5.9	48.7 ± 2.7	0.41
^4^ IL-10 UNS	33.1 ± 4.2	29.1 ± 1.8	0.49	28.3 ± 2.4	33.1 ± 3.1	0.30	29.2 ± 6.8	35.4 ± 5.6	27.9 ± 2.4	30.8 ± 2.8	0.70
TNFα ConA	773.6 ± 57	727.4 ± 31	0.43	779.1 ± 42	711.8 ± 41	0.30	792.9 ± 87	754.3 ± 79	770.0 ± 42	680.9 ± 43	0.68
TNFα UNS	ND	ND	/	ND	ND	/	ND	ND	ND	ND	/

Values are means (pg/mL) ± standard error. Abbreviations: Ginseng berry extract (GBE); Interleukin-2 (IL-2); concanvalin A (ConA); unstimulated (UNS); interferon-γ (IFNγ); interleukin-10 (IL-10); tumor necrosis factor α (TNFα); ND = not detectable. ^1^ IL-2 UNS 4 samples from sham/water, 2 from sham/GBE, 4 from MI/water, 6 from MI/GBE were below detection limit of 0.46 pg/Ml; ^2^ IFNγ ConA 1 outlier removed from sham/water group; ^3^ IL-10 ConA 2 samples from MI/water and 1 from sham/water were below detection limit of 19.4 pg/mL; ^4^ IL-10 UNS 5 samples from sham/water, 3 from sham/GBE, 5 from MI/water, 6 from MI/GBE were below detection limit of 19.4 pg/mL.
