# Parikh, M., et al. Ginseng Berry Extract Rich in Phenolic Compounds Attenuates Oxidative Stress but not Cardiac Remodeling post Myocardial Infarction. *Int. J. Mol. Sci.* 2019, *20*, 983

**DOI:** 10.3390/ijms20071756

**Published:** 2019-04-09

**Authors:** International Journal of Molecular Sciences Editorial Office

**Affiliations:** MDPI, St. Alban-Anlage 66, 4052 Basel, Switzerland; ijms@mdpi.com

We would like to change the affiliations of the authors in our published paper [1]; the correct version is as follows:

Mihir Parikh ^1,2,3,†^, Pema Raj ^1,3,†^, Liping Yu ^1,4^, Jo-Ann Stebbing ^1,4^, Suvira Prashar ^1,4^, Jay C. Petkau ^1,4^, Paramjit S. Tappia ^5^, Grant N. Pierce ^1,2,3^, Yaw L. Siow ^1,3,4^, Dan Brown ^1,4^, Heather Blewett ^1,3,4,6^ and Thomas Netticadan ^1,3,4,^*

^1^  Canadian Centre for Agri-Food Research in Health and Medicine, St. Boniface Hospital Albrechtsen Research Centre, Winnipeg, MB R2H2A6, Canada; mparikh@sbrc.ca (M.P.); praj@sbrc.ca (P.R.); lyu@sbrc.ca (L.Y.); jstebbing@sbrc.ca (J.-A.S.); sprashar@sbrc.ca (S.P.); jpetkau@sbrc.ca (J.C.P.); gpierce@sbrc.ca (G.N.P.); csiow@sbrc.ca (Y.L.S.); danielbrown500@gmail.com (D.B.); hblewett@sbrc.ca (H.B.)
^2^  Institute of Cardiovascular Sciences, St. Boniface Hospital Albrechtsen Research Centre, Max Rady College of Medicine, Rady Faculty of Health Sciences, University of Manitoba, Winnipeg, MB R2H2A6, Canada
^3^  Department of Physiology and Pathophysiology, University of Manitoba, Winnipeg, MB R3E0J9, Canada
^4^  Agriculture and Agri-Food Canada, Winnipeg, MB R2H2A6, Canada
^5^  Asper Clinical Research Institute & Office of Clinical Research, St. Boniface Hospital, Winnipeg, MB R2H2A6, Canada; ptappia@sbrc.ca
^6^  Department of Food and Human Nutritional Sciences, University of Manitoba, Winnipeg, MB R3T2N2, Canada

*  Correspondence: tnetticadan@sbrc.ca
^†^  These authors contributed equally to this work.

We apologize for any inconvenience caused to the readers and authors by this error. The change does not affect the scientific results. The article will be updated, and the original will remain on the article webpage.
